# The Stratifying Value of Hangzhou Criteria in Liver Transplantation for Hepatocellular Carcinoma

**DOI:** 10.1371/journal.pone.0093128

**Published:** 2014-03-27

**Authors:** Jun Chen, Xiao Xu, Jian Wu, Qi Ling, Kai Wang, Weilin Wang, Min Zhang, Yan Shen, Lin Zhou, Haiyang Xie, Shusen Zheng

**Affiliations:** 1 Division of Hepatobiliary and Pancreatic Surgery, Department of Surgery, First Affiliated Hospital, Zhejiang University School of Medicine, Hangzhou, Zhejiang Province, China; 2 Collaborative Innovation Center for Diagnosis and Treatment of Infectious Diseases, Hangzhou, Zhejiang Province, China; Xiangya Hospital of Central South University, China

## Abstract

**Background/Aims:**

The selection criteria for patients with hepatocellular carcinoma (HCC) as candidates for deceased donor liver transplantation (DDLT) are well studied. In this era of limited deceased donor organs, the value of living donor liver transplantation (LDLT) for HCC remains controversial. The aim of the present study was to verify the stratification value of the Hangzhou criteria for LDLT.

**Methods:**

The data of 47 LDLT recipients and 94 matched DDLT recipients at our center were evaluated. Overall survival and tumor-free survival were calculated. Prognostic factors influencing post-liver transplantation (LT) survival were identified. The stratification values of the Hangzhou criteria and Milan criteria were compared.

**Results:**

LDLT recipients spent much less time on the waiting list. The post-LT survival of recipients fulfilling the Milan criteria and recipients fulfilling the Hangzhou criteria were comparable (*P*>0.05). The overall and tumor-free survival did not differ statistically between the two groups. In both groups, more recipients not meeting the Milan criteria but with a satisfactory outcome were identified by the Hangzhou criteria. Among recipients who did not meet the Hangzhou criteria, tumor-free survival was better for the LDLT recipients than the DDLT recipients (*P* = 0.024).

**Conclusions:**

The Hangzhou criteria are reliable for stratifying HCC patients in terms of prognosis. HCC patients fulfilling the Hangzhou criteria gain satisfactory survival from LT. Outcomes after LDLT are better than those after DDLT for HCC patients who do not meet the Hangzhou criteria.

## Introduction

Hepatocellular carcinoma (HCC) is a common disease, with more than a half-million new cases occurring worldwide each year. This makes it the fifth most common neoplasm in the world and the third major cancer killer [1]. The neoplasm almost invariably arises in the setting of hepatitis B virus (HBV) or hepatitis C virus (HCV)-induced cirrhosis. Only surgical removal of the tumor offers a chance of cure or long-term survival. Because the procedure may remove both the tumor and the entire cirrhotic background, liver transplantation (LT) is widely accepted as the treatment of choice for cirrhosis-related HCC. It might be the only life-saving procedure for patients with unresectable HCC and end-stage liver disease. With exposure to immunosuppressive agents, the HCC recurrence is usually fiercer after LT than after partial liver resection in unselected patients [2]. The Milan criteria and UCSF criteria [3], [4] and the Hangzhou criteria [5] have been shown to be successful in improving the outcomes of deceased donor liver transplantation (DDLT) for HCC performed at different centers and in persons of different racial backgrounds [6]–[8]. Since the first successful living donor liver transplantation (LDLT) in 1998, LDLT has emerged as an alternative choice for treatment of HCC, particularly in the current era of limited supply of deceased donor organs. To the best of our knowledge, the establishment of reliable candidate selection criteria suitable for LDLT in patients with HCC is feasible, although it has not yet been done. Respecting this possibility, we retrospectively studied the cases of LDLT for HCC performed at our center with the goal of verifying the feasibility and selective value of the Hangzhou criteria in the context of LDLT.

## Materials and Methods

### Ethics statement

This study was approved by the First Affiliated Hospital, Zhejiang University School of Medicine. The current regulation of the Chinese Government and the Declaration of Helsinki were strictly followed for each organ donation and transplant performed at our center. Written informed consent from each donor and recipient were obtained. All data were analyzed anonymously.

### Patients

Between May 2007 and March 2012, 47 consecutive HCC-LDLTs (HCC was confirmed by histological examination of the explanted liver) were performed at the Liver Transplantation centerof the First Affiliated Hospital, Zhejiang University China. For comparison, 94 matched (a 1:2 match for both general information and tumor-related information) HCC related DDLT in the same center simultaneously were included in this study. These two groups of recipients are referred to as Group L (47 LDLT recipients) and Group D (94 matched DDLT recipients). Variables of the two groups were compared to determine differences in distribution. The data for both groups are shown in Table 1.

**Table 1:** Baseline characteristics of patients before transplantation (Chi-Square Tests).

Variables	Grading	LDLT (n = 47)	DDLT (n = 94)	*P* value
Age (year)	≤50	25	44	0.482
	>50	22	50	
Gender	male	44	88	1.000
	female	3	6	
Liver cirrhosis	with	46	88	0.424
	without	1	6	
Child-Pugh score	A(5–6)	19	42	0.886
	B(7–9)	18	34	
	C(≥10)	10	18	
Serum AFP level (ng/ml)	≤400	26	53	0.523
	>400	21	41	
Macrovascular invasion	with	6	12	1.000
	without	41	82	
Tumor differentiation	well/moderate	30	55	0.587
	poor	17	39	
Pre-LT partial hepatectomy	with	10	14	0.351
	without	37	80	
Milan criteria	fulfill	17	36	0.855
	exceed	30	58	
Hangzhou criteria	fulfill	26	52	0.858
	exceed	21	42	

### Post-transplantation management and follow up

The post-operation immunosuppressive protocols involved tacrolimus/ciclosporin or sirolimus, Mycophenolate mofetil and steroids (withdraw within the first month after LT). Follow-up was routinely performed in the out-patient clinics. Surveillance against recurrence comprised ultrasonography (performed monthly for the first half year, then quarterly), computed tomography (CT), and emission computed tomography (ECT) examinations (performed quarterly for the first year, biannually for the second year, then annually), and measurement of alpha-fetoprotein (AFP) (performed monthly for the first half year, bimonthly for the second half year, then biannually). Transplant recipients with HCC recurrence were referred for corresponding resection and other interventional treatments such as radiofrequency ablation (RFA), transarterial chemoembolization (TACE), percutaneous ethanol injection (PEI) and internal radiotherapy. The Post-transplantation managements and follow up strategies were mentioned in our previous study [5].

### Statistical analysis

Descriptive variables are expressed as mean±standard error and median values. Continuous data were compared by the 2-tailed Student’s t test. Overall survival (OS) and tumor-free survival (TFS) were calculated according to the Kaplan-Meier method and compared by the log-rank test. Tumor-free survival was calculated by including all recipients, whether deceased or living, with recurrence as the event. Constituent ratios of prognostic factors between groups were analyzed by theχ^2^ test. *P* <0.05 was considered statistically significant. The variables compared by univariate analysis in the present study included sex (male/female), age (<50 years/≥50 years), underlying liver cirrhosis (with/without), hepatectomy history (with/without), Child-Pugh classification (A/B/C), preoperative AFP level (≤20 ng/ml/20–400 ng/ml/*>*400 ng/ml), tumor differentiation (well-differentiated/ moderately differentiated/ poorly differentiated), macrovascular invasion (with/without) (most of which were found as tumor thrombi in small branches of the portal or hepatic vein and confirmed by the pathologic examination of explants but not detected by pre-LT imaging study), the Hangzhou criteria (a maximum tumor diameter of no more than 8 cm or a maximum tumor diameter of more than 8 cm, with well or moderately differentiated, and with a preoperative AFP level of no more than 400 ng/ml) [5] and the Milan criteria (a single lesion not larger than 5 cm or 2 or 3 lesions not larger than 3 cm each) (within/exceed). These variables were analyzed to evaluate their determinant value for post-LT survival. The variables shown to be statistically significant in univariate analysis were entered into forward step-wise multivariate Cox proportional hazards analysis to evaluate the relative risk (RR) for post-LT survival. All data were analyzed statistically with the use of SPSS, version 11.0 (SPSS Inc, Chicago, IL). The statistical methods mentioned above were similar to that of our previous study [5].

## Results

### Survival

Patients were followed up for a mean 1246 days (range, 43– 2710 days). The mean time on the waiting list for Group L was 22 days, which was significantly shorter than that for Group D (91 days, *P*<0.001). The post-LT survival (OS and TFS) was comparable between recipients fulfilling the Milan criteria and recipients fulfilling the Hangzhou criteria (*P*>0.05). In Group L, the 1- and 5-year OS and the 1- and 5-year TFS of recipients fulfilling the Hangzhou criteria (n = 26) were 100%, 87.7%, and 96.2%, 80.3% (*P*<0.001) respectively; those of recipients not fulfilling the Hangzhou criteria were 71.4%, 33.3%, and 42.9%, 28.6%, respectively, (n = 21, *P*<0.001). In Group D, the 1- and 5-year OS and 1- and 5-year TFS of recipients fulfilling the Hangzhou criteria (n = 52) were 92.3%, 80.8% and 92.3%, 76.1%, respectively, those of recipients not fulfilling the Hangzhou criteria were 59.5%, 11.9% and 21.4%, 6.3%, respectively (n = 42, both *P*<0.001). The 1- and 5-year OS and 1- and 5-year TFS of recipients fulfilling the Milan criteria in Group L (n = 17) and Group D (n = 36) were also significantly better than those of the patients not fulfilling the Milan criteria (*P*<0.001).

### Recurrence and metastasis

Post-LT tumor recurrence or metastasis developed in 68 recipients (48.2%), 19 in Group L and 49 in Group D. The incidence of HCC recurrence in the two groups was comparable (*P*>0.05). Tumor recurrence was the main cause of post-LT death (93.8%). The mean time between transplantation and the diagnosis of HCC recurrence or metastasis was comparable between Group L and Group D (400±385 days and 267±215 days, respectively, *P*>0.05). The liver graft, lung, and bone were the predominant sites of recurrence or metastasis. All recipients with recurrence were referred for surgical intervention and medical treatment.

### Risk factors for post-LT survival

According to the univariate analysis, six variables, the preoperative AFP level, fulfilment of the Milan criteria, fulfilment of the Hangzhou criteria, tumor differentiation, macrovascular invasion, and underlying liver cirrhosis (only for overall survival) were significant prognosticators for survival in Group D. The same variables, with the exception of underlying liver cirrhosis were also significant prognosticators for survival in Group L (Table 2). All the identified dependent risk factors of survival in both groups were then sent to the multivariate Cox regression analysis.

**Table 2:** Univariate analysis of variables related to post-LT survival (log rank test).

Variables	Post-LT survival	*P* value	
		Group L	Group D
Age (year)(≤50 *VS.* >50)	overall survival tumor-free survival	0.5030.606	0.1230.088
Gender(male *VS.* female)	overall survival tumor-free survival	0.8100.625	0.1440.109
Liver cirrhosis(with *VS.* without)	overall survival tumor-free survival	0.3360.353	0.029*0.071
Child-Pugh score(A *VS.* B *VS.* C)	overall survival tumor-free survival	0.1110.273	0.7970.716
Serum AFP level (ng/ml)(≤400 *VS.* >400)	overall survival tumor-free survival	0.045*0.003*	<0.001*<0.001*
Macrovascular invasion(with *VS.* without)	overall survival tumor-free survival	<0.001*0.001*	0.002*0.001*
Tumor differentiation(well/ moderate *VS.* poor)	overall survival tumor-free survival	0.005*0.024*	0.030*0.002*
Pre-LT partial hepatectomy(with *VS.* without)	overall survival tumor-free survival	0.2360.536	0.7610.736
Milan criteria(fulfill *VS.* exceed)	overall survival tumor-free survival	0.011*0.007*	<0.001*<0.001*
Hangzhou criteria(fulfill *VS.* exceed)	overall survival tumor-free survival	<0.001*<0.001*	<0.001*<0.001*

*: sent to the multivariate Cox regression analysis.

According to the Cox regression analysis, fulfilling the Hangzhou criteria was a significant independent variable for survival in both Group D and Group L. Fulfilling the Milan criteria was shown to be a significant independent variable for survival in Group D but not in Group L (Table 3).

**Table 3:** Independent variables in the Cox analysis for post-LT survival (Forward test).

	Variables	Overall survival Relative risk *P* value		Tumor-free survival Relative risk *P* value	
Group D	Fulfill Hangzhou criteria	3.287	0.004	5.126	<0.001
	Fulfill Milan criteria	7.818	0.012	3.477	0.044
Group L	Fulfill Hangzhou criteria	9.157	0.001	6.192	<0.001

In order to analyze the value of Hangzhou criteria for stratifying HCC patients, other important variables such as Child-Pugh score and MELD score who do not meet the Hangzhou criteria or Milan criteria were also analyzed. Different Child-Pugh score and MELD score didn’t show any correlation with fulfilling Hangzhou/ Milan criteria or not.

### LDLT versus DDLT

**Figure 1:**
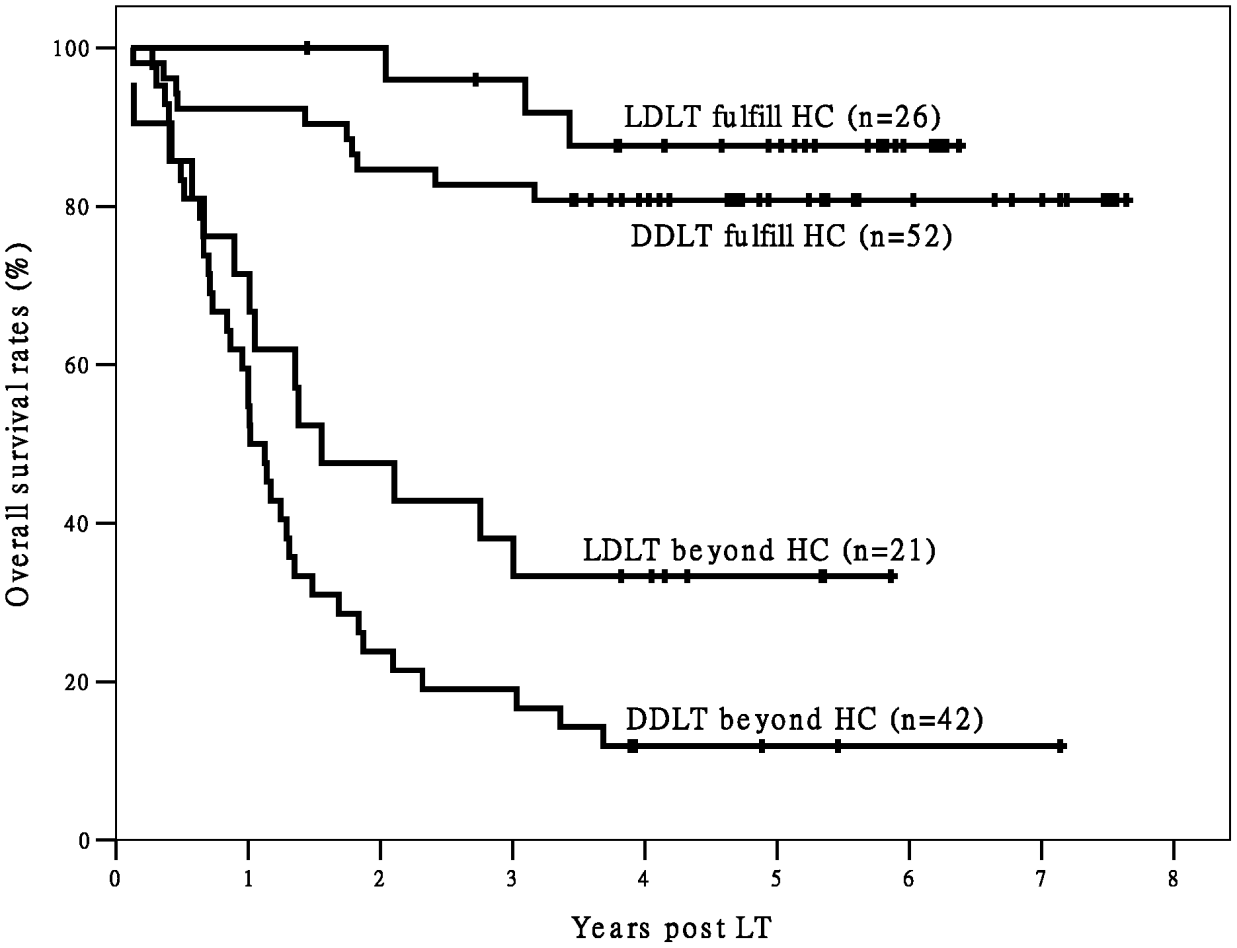
The survival curves of post-LT OS of recipients. There is a significant difference in OS between recipients fulfilling Hangzhou criteria and patients exceeding Hangzhou criteria (P<0.05). There is no significant difference in OS between different transplant types within Hangzhou criteria (P>0.05). There is also no significant difference in OS between different transplant types beyond Hangzhou criteria (P = 0.065). Abbreviation: OS, overall survival; HC, Hangzhou criteria; LDLT, living donor liver transplantation; DDLT, deceased donor liver transplantation; LT, liver transplantation;

**Figure 2:**
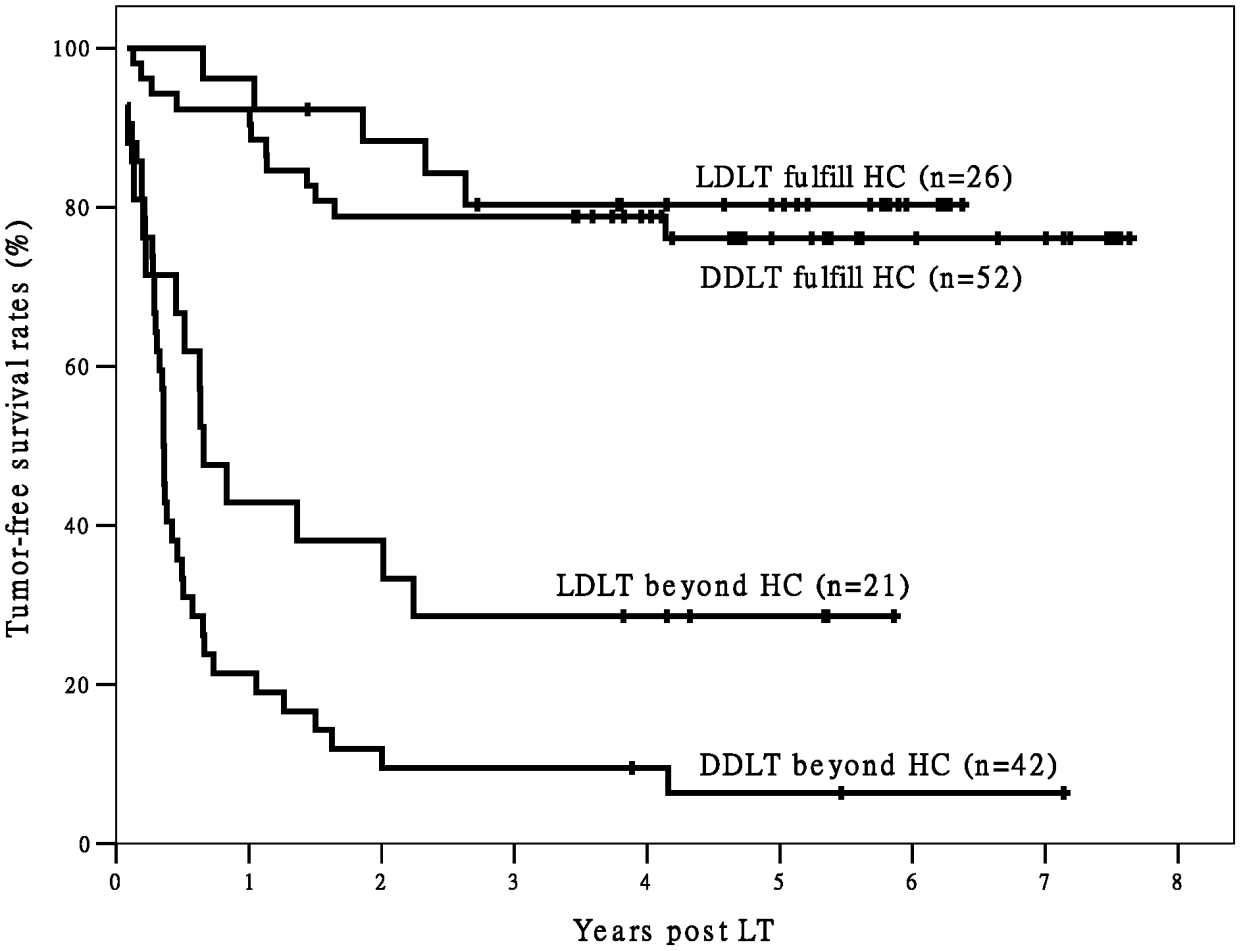
The survival curves of post-LT TFS of recipients. There is a significant difference in TFS between recipients fulfilling Hangzhou criteria and patients exceeding Hangzhou criteria (P<0.05). There is no significant difference in TFS between different transplant types within Hangzhou criteria (P>0.05). However, there is significant difference in TFS between different transplant types beyond Hangzhou criteria (P = 0.024). Abbreviation: TFS, tumor-free survival; HC, Hangzhou criteria; LDLT, living donor liver transplantation; DDLT, deceased donor liver transplantation; LT, liver transplantation;

When we divided Group L and Group D into two subgroups each (for a total of four subgroups) according to fulfillment/non-fulfillment of the Hangzhou criteria, the differences in post-LT OS and TFS between the two subgroups fulfilling Hangzhou criteria and the other two subgroups were statistically significant (Figures 1 and 2). Only three deaths were observed among recipients fulfilling the Hangzhou criteria in Group L (n = 26) during the follow-up period. The Group L recipients fulfilling the Hangzhou criteria achieved good post-LT survival. In addition, the recipients in Group L that did not meet the Hangzhou criteria had significantly better TFS (*P* = 0.024) and better, but not significantly better OS (*P* = 0.065) than recipients in Group D who did not meet the Hangzhou criteria.

### Hangzhou criteria versus Milan criteria in LDLT

**Figure 3:**
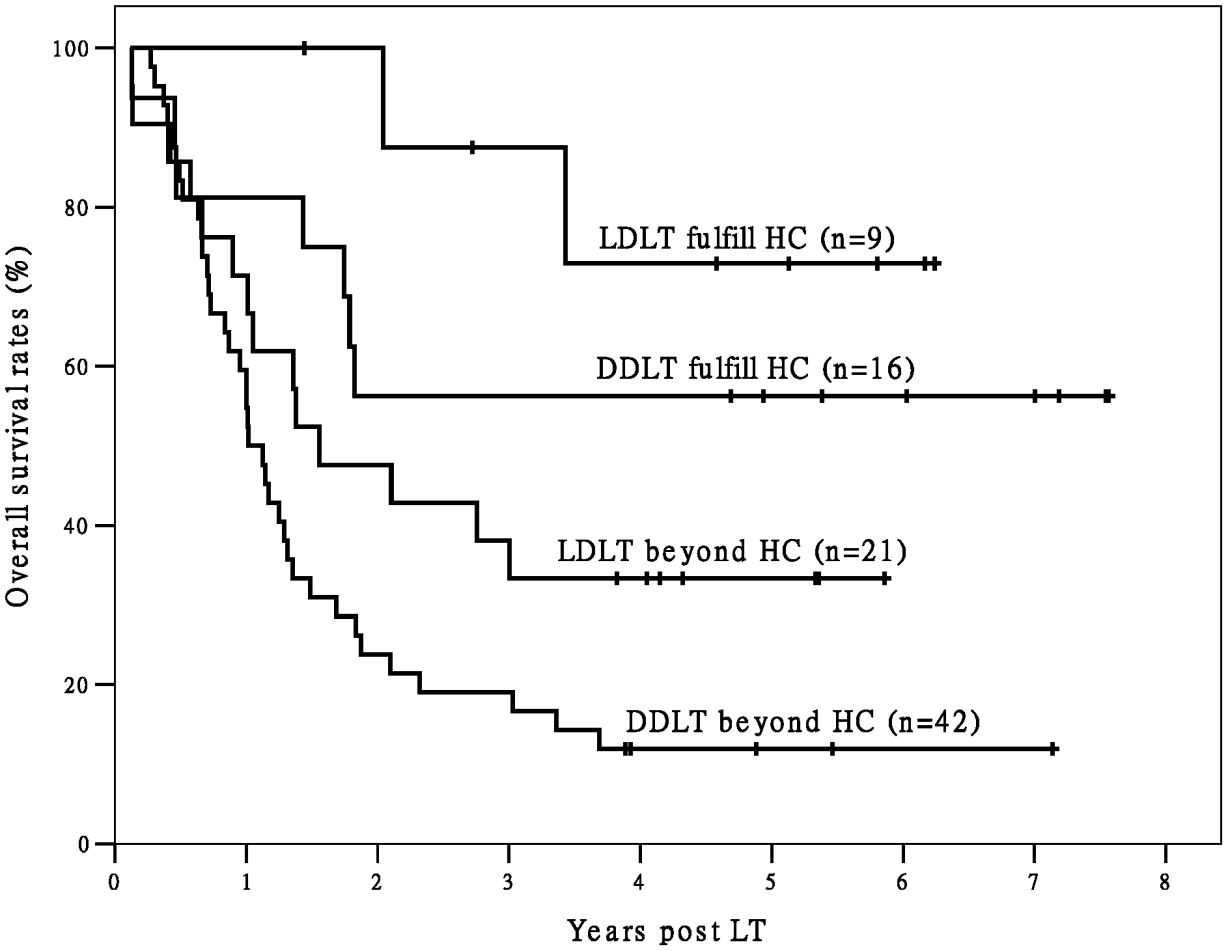
The survival curves of post-LT OS of recipients beyond Milan criteria. There are significant differences in OS between LDLT/ DDLT recipients fulfilling Hangzhou criteria and LDLT/ DDLT recipients exceeding Hangzhou criteria in all recipients beyond Milan criteria (both P<0.05). Abbreviation: HC, Hangzhou criteria; OS, overall survival; LDLT, living donor liver transplantation; DDLT, deceased donor liver transplantation;

**Figure 4:**
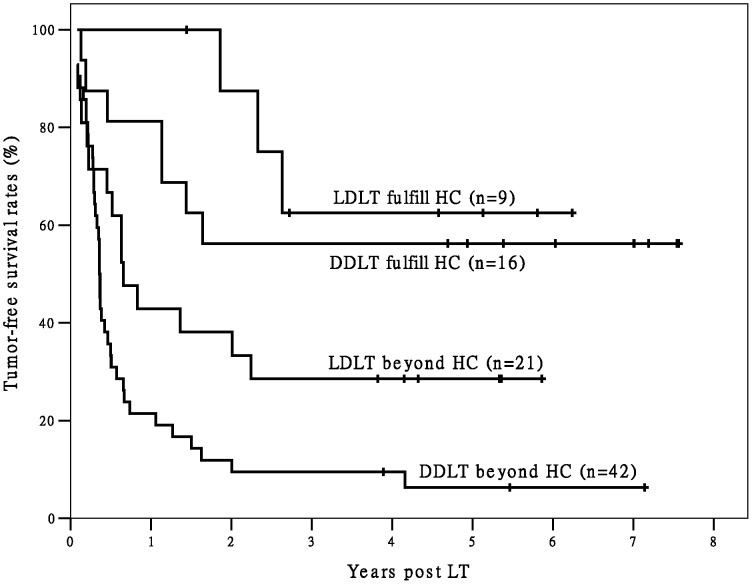
The survival curves of post-LT TFS of recipients beyond Milan criteria. There are significant differences in TFS between LDLT/ DDLT recipients fulfilling Hangzhou criteria and LDLT/ DDLT recipients exceeding Hangzhou criteria in all recipients beyond Milan criteria (both P<0.05). Abbreviation: HC, Hangzhou criteria; TFS, tumor-free survival; LDLT, living donor liver transplantation; DDLT, deceased donor liver transplantation;

**Figure 5:**
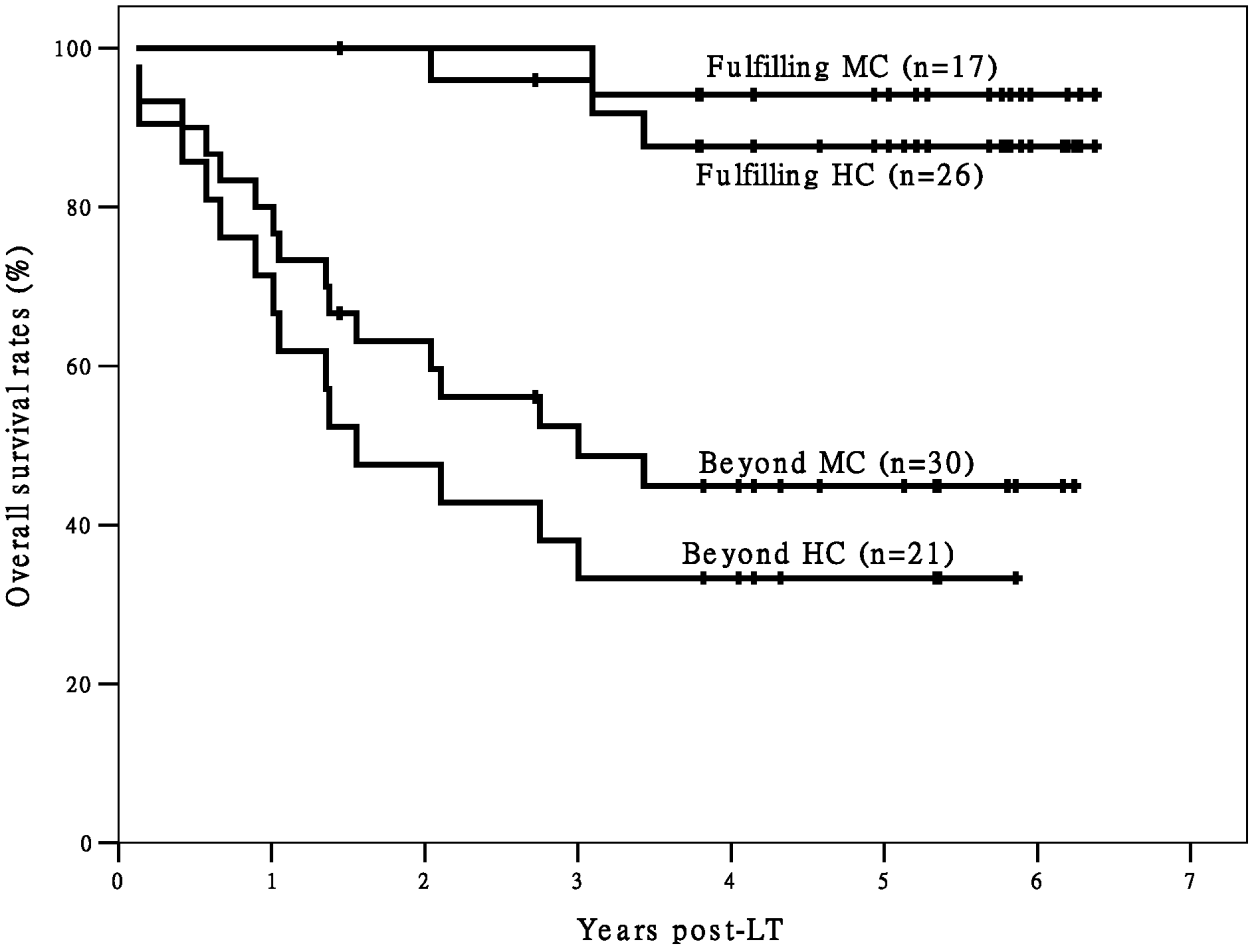
The survival curves of post-LT OS of Milan criteria and Hangzhou criteria in LDLT. There is a significant difference in OS between recipients fulfilling Hangzhou/Milan criteria and patients exceeding Hangzhou/Milan criteria (P<0.05). There is no significant difference in OS between recipients fulfilling Milan criteria and recipients fulfilling Hangzhou criteria (P = 0.502).There is also no significant difference in OS between recipients beyond Milan criteria and recipients exceeding Hangzhou criteria (P = 0.330). Abbreviation: MC, Milan criteria; HC, Hangzhou criteria; OS, overall survival; LT, liver transplantation;

**Figure 6:**
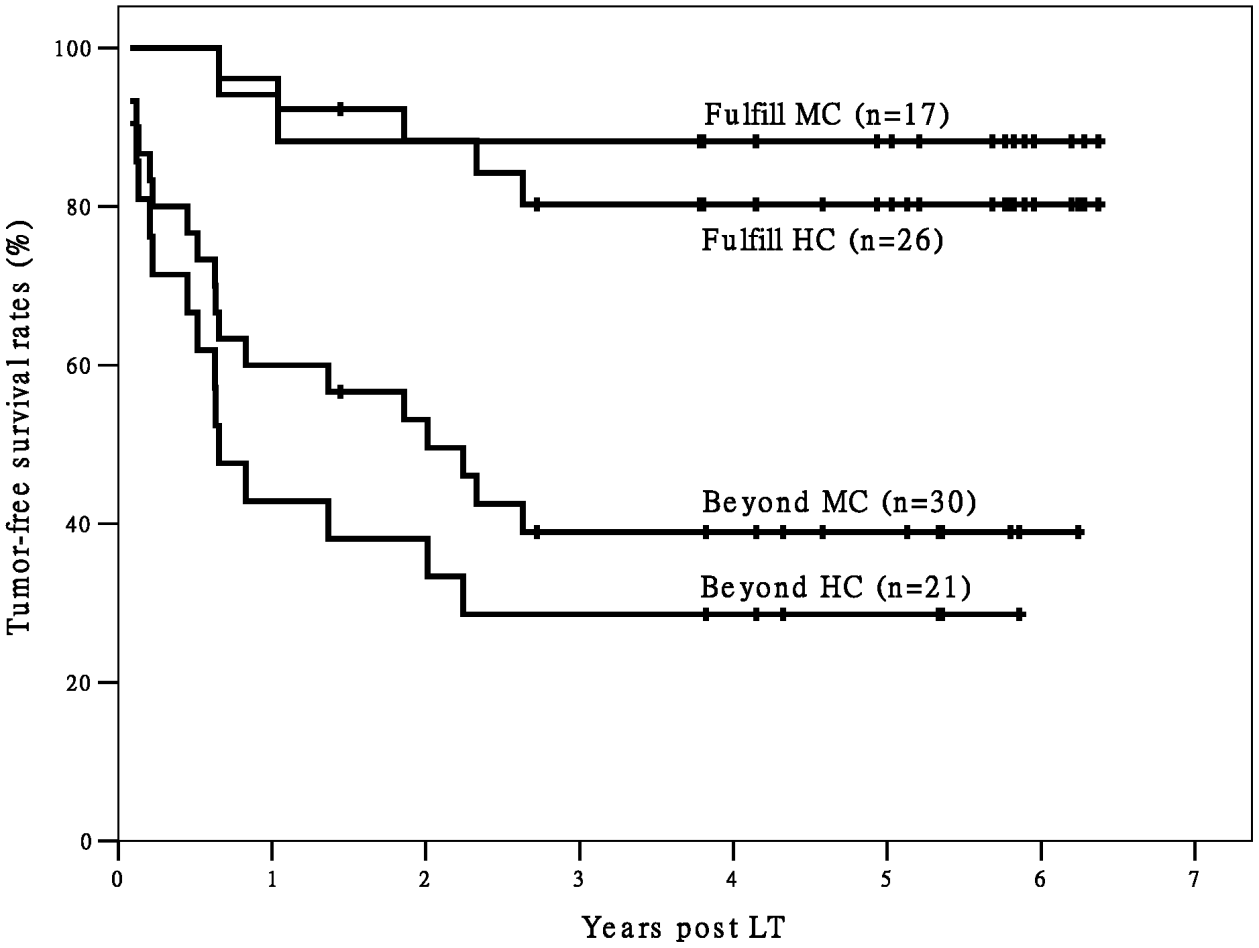
The survival curves of post-LT TFS of Milan criteria and Hangzhou criteria in LDLT. There is a significant difference in TFS between recipients fulfilling Hangzhou/Milan criteria and patients exceeding Hangzhou/Milan criteria (P<0.05). There is no significant difference in TFS between recipients fulfilling Milan criteria and recipients fulfilling Hangzhou criteria (P = 0.557).There is also no significant difference in TFS between recipients beyond Milan criteria and recipients beyond Hangzhou criteria (P = 0.289). Abbreviation: MC, Milan criteria; HC, Hangzhou criteria; TFS, tumor-free survival; LT, liver transplantation;

In Group L there were 17 recipients with good outcome were identified by the Milan criteria. Among the recipients not meeting the Milan criteria in Group L, those fulfilling the Hangzhou criteria (n = 9) also achieved better OS (*P* = 0.032) and TFS (*P* = 0.029) than the others (n = 21) (The Hangzhou criteria were also able to identify recipients not meeting the Milan criteria but having favorable post-LT outcomes in Group D, Figures 3 and 4). That means comparing with Milan criteria, Hangzhou criteria identified 53.0% (9/17) more recipients with excellent outcome. In Group L, recipients fulfilling the Hangzhou or Milan criteria had significantly better OS and TFS than recipients not meeting the Hangzhou or Milan criteria (both *P*<0.05). The OS and TFS of recipients fulfilling the Milan criteria and recipients fulfilling the Hangzhou criteria were comparable. The OS and TFS of recipients not meeting the Milan criteria and recipients not meeting the Hangzhou criteria were also comparable (Figures 5 and 6).

## Discussion

LDLT for the treatment of HCC remains a matter of concern. LDLT represents an alternative approach to HCC, which, in comparison to DDLT, allows for LT within a short time because grafts are not obtained from the limited cadaveric donor pool. Presently, the time spent on the waiting list for DDLT is much longer than that for LDLT (analogous to the present study), and the prolonged waiting time leads to the drop out at least 20-30% of candidates before LT, due to tumor progression [9], [10]. In an LDLT program, the much shorter hot/cold ischemic time that the liver graft is exposed to ensures better recovery of the graft function. However, the high rate of HCC recurrence has dampened the enthusiasm for LDLT as a treatment for HCC [11]-[13]. Moreover, because the graft donation in an LDLT program depends mainly on the donors’ voluntary wills, it is possible for advanced HCC patients, who are put at a disadvantage by the allocation algorithm for liver grafts from deceased donors, to have a chance to be cured by LDLT. Thus, the eligibility criteria for LDLT in HCC patients should be carefully investigated.

Since the worldwide acceptance of the Milan criteria for discriminating HCC patients for whom LT is indicated, the prevalence of LT has progressively increased. Several studies [14]–[17] confirmed that the Milan criteria and the UCSF criteria were both applicable to LDLT and DDLT. However, we proposed our novel candidate selection criteria for HCC-DDLT, which we referred to in a previous report as the Hangzhou criteria. The criteria were able to identify recipients not meeting the Milan criteria but having favorable post-LT outcomes. The feasibility of applying the Hangzhou criteria to HCC-DDLT has been evaluated and accepted by international LT experts [6]–[8]. Hence, the feasibility of applying the Hangzhou criteria to LDLT has deserved further investigation. In the present study, LDLT recipients fulfilling the Milan criteria and recipients fulfilling the Hangzhou criteria both achieved favorable OS; the TFS of these two types of recipients was also comparable. Moreover, the Hangzhou criteria can identify 53.0% more recipients who will have a favorable outcome than can the Milan criteria in an LDLT program. Recipients in the present study who did not meet the Hangzhou criteria achieved a better outcome after undergoing LDLT than after undergoing DDLT. LDLT for HCC has arisen to overcome the scarcity of deceased donor livers. Because living donor liver graft is a private gift directed to a particular beloved person, the decision for LDLT in HCC patients is based on the balance between risks and benefits for both the donor and recipient [18]. The special relationship between the donor and recipient may provide a recipient with the opportunity to undergo LDLT even in the event of advanced HCC. LDLT is a serious family matter that involves both the donor and recipient and does not jeopardize the limited supply of donated deceased donor organs, which constitutes a public resource [19]. Even if prospective LDLT candidates are not contraindicative, their situation must be discussed on a case-by-case basis, taking into account the presence of risk factors for recurrence and the wishes of the patient and family, especially when the family strongly requests LDLT for an advanced HCC patient [20]. Of course, this should also be involved with minimal donor morbidity and mortality.

Whether there is more frequent HCC recurrence after LDLT than after DDLT is the focus of the HCC-LDLT controversy. Previous studies attributed the poorer results of HCC-LDLT to the following three causes. First, with the shorter waiting time, LDLT may neither provide adequate time to assess a tumor’s aggressiveness nor allow clinically undetectable micrometastases or vascular invasion to become apparent [11], [12]. However, it can also be argued that placing patients with HCC on a fast track to transplant may reduce the chances of extrahepatic dissemination [21]. In the present study, no difference was observed in the rate of recurrence between the two types of recipients. Furthermore, among the recipients with recurrent HCC, the interval between LT and the diagnosis of HCC recurrence was comparable between LDLT recipients and DDLT recipients. Second, tumor growth induced by the up-regulation of factors during the natural course of liver regeneration of the partial liver graft may increase recurrence of HCC in LDLT patients [12], [15]. However, in the present study, the recurrences in the LDLT group occurred relatively late in comparison to the recurrences in the DDLT group, and LDLT has not been shown to be an independent risk factor for post-LT HCC recurrence (analogous to the present study [22]). Third, the dissection of the recipient liver is more meticulous for LDLT than for DDLT, with preservation of the native vena cava and greater hepatic artery and bile duct length. It is possible that LDLT is therefore a less optimal cancer operation, as it may leave residual tumor or violate the tumor capsule. Greater manipulation of the native liver may also lead to tumor embolus detachment through the hepatic veins. The technical requirements of the LDLT procedure may influence tumor recurrence to a greater degree [12], [23]. Actually, the liver hilum and retrohepatic area have never been shown to be predominant sites of recurrence in recipients of a living donor graft [21]. In the present study, there was no difference in the incidence of HCC recurrence between the two types of recipients. Moreover, none of the recipients in Group L had HCC recurrence in the hilum or in the area of the preserved native vena cava. However, these three arguments may explain the trend toward extrahepatic HCC metastasis in the LDLT group. Further study at the molecular or genetic level is needed.

In conclusion, the results of the present study reveal that the Hangzhou criteria are reliable in stratifying HCC patients in relation to different prognoses. HCC patients fulfilling the Hangzhou criteria gain satisfactory survival. HCC patients who do not meet the Hangzhou criteria can achieve a better outcome by LDLT than by DDLT. The importance of the AFP level should be emphasized in this LT era. Further, well-designed and prospective multicentre studies are needed to get the bottom of HCC recurrence in LDLT programs.
